# Discovering Panel of Autoantibodies for Early Detection of Lung Cancer Based on Focused Protein Array

**DOI:** 10.3389/fimmu.2021.658922

**Published:** 2021-04-23

**Authors:** Di Jiang, Xue Zhang, Man Liu, Yulin Wang, Tingting Wang, Lu Pei, Peng Wang, Hua Ye, Jianxiang Shi, Chunhua Song, Kaijuan Wang, Xiao Wang, Liping Dai, Jianying Zhang

**Affiliations:** ^1^Department of Oncology, Henan Institute of Medical and Pharmaceutical Sciences, Zhengzhou University, Zhengzhou, China; ^2^School of Basic Medical Sciences, Academy of Medical Science, Zhengzhou University, Zhengzhou, China; ^3^Henan Key Laboratory of Tumor Epidemiology & State Key Laboratory of Esophageal Cancer Prevention, Zhengzhou University, Zhengzhou, China; ^4^Department of Clinical Laboratory, Fuwai Central China Cardiovascular Hospital, Zhengzhou, China; ^5^Department of Clinical Laboratory, Zhengzhou Hospital of Traditional Chinese Medicine, Zhengzhou, China; ^6^Department of Epidemiology and Biostatistics in School of Public Health, Zhengzhou University, Zhengzhou, China

**Keywords:** lung cancer, protein array, tumor-associated antigen, autoantibody, diagnostic model

## Abstract

Substantial studies indicate that autoantibodies to tumor-associated antigens (TAAbs) arise in early stage of lung cancer (LC). However, since single TAAbs as non-invasive biomarkers reveal low diagnostic performances, a panel approach is needed to provide more clues for early detection of LC. In the present research, potential TAAbs were screened in 150 serum samples by focused protein array based on 154 proteins encoded by cancer driver genes. Indirect enzyme-linked immunosorbent assay (ELISA) was used to verify and validate TAAbs in two independent datasets with 1,054 participants (310 in verification cohort, 744 in validation cohort). In both verification and validation cohorts, eight TAAbs were higher in serum of LC patients compared with normal controls. Moreover, diagnostic models were built and evaluated in the training set and the test set of validation cohort by six data mining methods. In contrast to the other five models, the decision tree (DT) model containing seven TAAbs (TP53, NPM1, FGFR2, PIK3CA, GNA11, HIST1H3B, and TSC1), built in the training set, yielded the highest diagnostic value with the area under the receiver operating characteristic curve (AUC) of 0.897, the sensitivity of 94.4% and the specificity of 84.9%. The model was further assessed in the test set and exhibited an AUC of 0.838 with the sensitivity of 89.4% and the specificity of 78.2%. Interestingly, the accuracies of this model in both early and advanced stage were close to 90%, much more effective than that of single TAAbs. Protein array based on cancer driver genes is effective in screening and discovering potential TAAbs of LC. The TAAbs panel with TP53, NPM1, FGFR2, PIK3CA, GNA11, HIST1H3B, and TSC1 is excellent in early detection of LC, and they might be new target in LC immunotherapy.

## Introduction

Lung cancer (LC) is one of the leading causes of cancer-related deaths worldwide, accounting for 28% of all cancer deaths (1, 2). In China, LC is the first common cause of cancer-related death in men and the second cause in women (3). Due to the lack of effective early diagnosis technology for LC, it remains a challenge to improve the overall survival of patients with LC (4, 5). In the past 50 years, the 5-year survival rate of LC patients at early stage is 60–70%, while it is dreadfully < 5% at late stage (3). Therefore, early diagnosis is a critical factor to reduce the mortality and improve the long-term survival rate of LC patients (6, 7). Low-dose computed tomography (LDCT) emerged as a novel screening method for LC in 1990's, it was reported with 20% reduction of LC-related death in National Lung Cancer Screening Trial (NLST) by LDCT (8). Nevertheless, LDCT has up to 90% false-positive rate, thus it is necessary to confirm the diagnosis by additional invasive surgery or repeated radiation exposure (9), which bring unnecessary burden to the patient's economy and body.

Blood tumor biomarkers are potential for early diagnosis of LC as they have advantages of non-invasion and convenient to access (10, 11). However, multiple tumor biomarkers utilized in clinical practice show low diagnostic accuracy for cancer, such as carcinoembryonic antigen (CEA), neuron-specific enolase (NSE), and cytokeratin-19 fragment (CYFRA 21-1) (12–14). Tumor-associated antigens (TAAs) refer to antigen molecules that exist on tumor cells or normal cells, but they are abnormally expressed in diverse cancers (15). Autoantibodies to TAAs (TAAbs) are produced in early stage of cancers by humoral immune response triggered by abnormal expression of TAAs. In comparison with other types of biomarkers, serum TAAbs appeared earlier and more stable (16). They are a kind of promising biomarkers which could be applied for early diagnosis in cancers (17).

Recently, the protein array technology was commonly applied in identifying new TAAbs, which can simultaneously analyze large number of proteins in parallel and recognize posttranslational modified proteins (18, 19). The mutation of cancer driver genes may be one of the important factors for the occurrence of cancers (20). Based on the 138 cancer driver genes (74 tumor suppressor genes and 64 oncogenes) listed in study of Vogelstein et al. (21), we customized a protein array with 154 human recombinant proteins to explore the autoantibodies against TAAs in LC. The selected TAAbs were further validated by enzyme-linked immunosorbent assay (ELISA). Since single TAAb was limited by low sensitivity and accuracy and combined multiple TAAbs could improve the detection rate of LC effectively (22–24), a series of data mining techniques were performed to establish diagnostic models for LC, such as logistic regression, Fisher discriminate analysis, decision tree (DT), support vector machines (SVM), artificial neural network-multilayer perception (ANN-MLP), and artificial neural network-radial basis function (ANN-RBF). Finally, we evaluated the diagnostic efficacy of these models and chose DT model as the optimal model.

## Materials and Methods

### Study Populations

In this study, totally 1,204 subjects [555 LCs, 505 normal controls (NCs), and 144 benign lung disease cases (BLDs)] in three independent cohorts (discovery cohort, verification cohort, and validation cohort) were recruited from the First Affiliated Hospital of Zhengzhou University in Henan province, China between November 2016 and April 2019 (Table 1). All specimens were collected with patients' written informed consent, and the study protocol was approved by Medical Ethics Committee of Zhengzhou University (Zhengzhou, China). The process of serum specimen preparation and the inclusion criteria of subjects were presented in Supplementary Texts 1,2, respectively.

**Table 1:** Characteristics of populations in this study.

	**Discovery cohort**		**Verification Cohort**		**Validation Cohort**		
	**LC**	**NC**	**LC**	**NC**	**LC**	**BLD**	**NC**
	**N (%)**	**N (%)**	**N (%)**	**N (%)**	**N (%)**	**N (%)**	**N (%)**
***N***	100	50	155	155	300	144	300
**Age**							
Mean ± SD (years)	61 ± 11	40 ± 13	61 ± 10	60 ± 11	61 ± 11	60 ± 10	57 ± 11
Range (years)	26–85	20–71	30–83	28–81	26–87	29–85	25–89
**Gender**							
Male	66 (66.0)	23 (46.0)	116 (74.8)	116 (74.8)	185 (61.7)	103 (71.5)	156 (52.0)
Female	34 (34.0)	27 (54.0)	39 (25.2)	39 (25.2)	115 (38.3)	41 (28.5)	144 (48.0)
**Smokers**							
Yes	45 (45.0)		98 (63.2)		111 (37.0)	78 (54.2)	
No	55 (55.0)		57 (36.8)		178 (59.3)	66 (45.8)	
Unknown	0 (0.0)		0 (0.0)		11 (3.7)	0 (0.0)	
**Drinkers**							
Yes	26 (26.0)		45 (29.0)		54 (18.0)	36 (25.0)	
No	74 (74.0)		110 (71.0)		233 (77.7)	108 (75.0)	
Unknown	0 (0.0)		0 (0.0)		13 (4.3)	0 (0.0)	
**Family history of tumor**							
Yes	12 (12.0)		28 (18.1)		22 (7.3)	18 (12.5)	
No	88 (88.0)		127 (81.9)		263 (87.7)	126 (87.5)	
Unknown	0 (0.0)		0 (0.0)		15 (5.0)	0 (0.0)	
**Clinical stage**							
Stage I	18 (18.0)		11 (7.1)		51 (17.0)		
Stage II	12 (12.0)		11 (7.1)		12 (4.0)		
Stage III	33 (33.0)		58 (37.4)		44 (14.7)		
Stage IV	37 (37.0)		60 (38.7)		81 (27.0)		
Unknown	0 (0.0)		15 (9.7)		112 (37.3)		
**Histological type**							
SCC	31 (31.0)		42 (27.1)		64 (21.3)		
AD	68 (68.0)		58 (37.4)		177 (59.0)		
SCLC	0 (0.0)		43 (27.7)		32 (10.7)		
Others	1 (1.0)		12 (7.8)		15 (5.0)		
Unknown	0 (0.0)		0 (0.0)		12 (4.0)		
**Tumor size**							
≤ 5 cm	60 (60.0)		59 (38.1)		126 (42.0)		
>5 cm	40 (40.0)		80 (51.6)		79 (26.3)		
Unknown	0 (0.0)		16 (10.3)		95 (31.7)		
**Lymph node metastasis**							
Yes	69 (69.0)		99 (63.9)		124 (41.3)		
No	31 (31.0)		41 (26.4)		72 (24.0)		
Unknown	0 (0.0)		15 (9.7)		104 (34.7)		
**Distant metastasis**							
Yes	38 (38.0)		61 (39.4)		109 (36.3)		
No	62 (62.0)		79 (50.9)		112 (37.4)		
Unknown	0 (0.0)		15 (9.7)		79 (26.3)		
**Benign disease type**							
COPD						72 (50.0)	
Chronic bronchitis						72 (50.0)	

*AD, adenocarcinoma; BLD, benign lung disease; COPD, chronic obstructive pulmonary disease; LC, lung cancer; NC, normal control; SCC, squamous cell carcinoma; SCLC, small cell lung cancer; SD, standard deviation*.

### Focused Protein Array

A total of 154 human source recombinant proteins, including 143 proteins encoded by cancer driver genes and 11 proteins (CyclinB1, c-Myc, CIP2A/p90, IMP1, IMP2, IMP3, RalA, RBM39, YWHAZ, and two fragments of Survivin) previously researched in our laboratory, were contained in the focused protein array. The array was customized in CDI Laboratories (Mayaguez, USA). The array screening, data extraction, and analysis were implemented according to the protocol illustrated in Supplementary Text 3. Signal-to-noise ratio (SNR) was used to describe the serum level of autoantibodies in the subjects of discovery cohort. Based on the results of array test, we carried out comprehensive analyses to screen candidate TAAbs for LC (Supplementary Figure 1).

### ELISA

Indirect ELISA was used to detect the level of candidate TAAbs in serum samples of verification cohort and validation cohort. Detailed steps of the indirect ELISA experiment are presented in Supplementary Text 4. In this study, the verification cohort was used to test the eligibility of candidate TAAbs, and validation cohort to further validate the diagnostic performance of TAAbs. The positive and negative control sera of the TAAb were set in each plate for quality control. Furthermore, the concentration of autoantibodies in the serum was calculated according to the IgG standard curve of each plate.

### The Establishment of Diagnostic Model by Data Mining Methods

All diagnostic models were established by using SPSS Modeler 18.0 software. In order to establish and externally evaluate the diagnostic models, all LCs and NCs in the validation cohort were randomly divided into training (*N* = 414) and test (*N* = 186) sets according to the proportion of 7:3 by SPSS 21.0 software. Logistic regression analysis, Fisher discriminant analysis, DT C5.0, SVM, ANN-MLP, and ANN-RBF were applied to build models based on training set and then the models' performance were validated in test set. Additionally, Logistic regression models were established through forward and backward conditional logistic regression, respectively. The stepwise method and internal cross-validation were used in the Fisher discriminant model. In the construction of DT C5.0 model, decision tree was picked as the model output type with 10-fold cross-validation as internal validation. In order to improve the model, expert and global pruning mode were chosen, meanwhile, pruning severity and the minimum number of record for each sub-branch were set to 80 and 2, respectively. We also constructed models by MLP and RBF methods. MLP had more terminative rules than RBF (using a maximum training time of 1 min) and overfitting prevents the set from being 50.0% when choosing parameters of model. Moreover, we established SVM model in which the expert mode was selected. All methods were applied to distinguish LCs from NC.

### Statistical Analysis

SPSS 21.0 software package, GraphPad Prism 5.0, and MedCalc 11 were used to analyze and visualize the data from ELISA in this research. Differences of TAAbs levels among the different groups were analyzed by non-parametric tests and Wilcoxon test with Bonferroni adjustment. The sensitivity, specificity, and AUC with 95% confidence internal (CI) were all calculated by receiver operating characteristic (ROC) curve analysis. The OD value produced at the highest Youden's Index (sensitivity + specificity −1) was set as the cutoff value. The difference was considered statistically significant while *P* < 0.05.

## Results

### Overall Study Design

**Figure 1:**
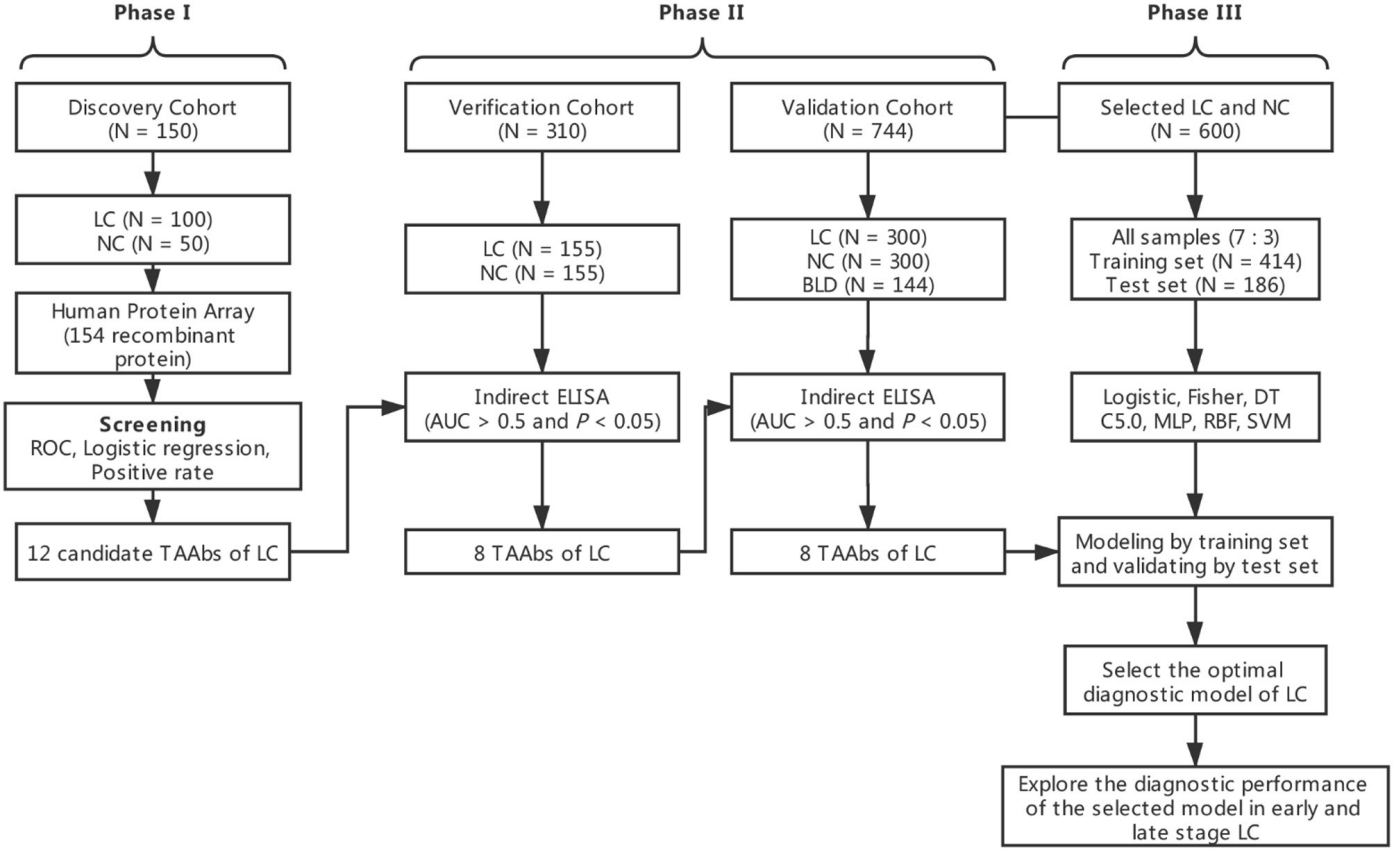
Overall study design.

The overall study was divided into three phases including the discovery of potential TAAbs, the validation of candidate TAAbs, and the establishment of diagnostic models (Figure 1). Briefly, in phase I, the serum samples of discovery cohort containing 100 LCs and 50 NCs were individually profiled on focused protein array. In phase II, 155 LCs and 155 NCs in the verification cohort were matched by age and gender, which was used to verify the screened candidate TAAbs from protein array. In addition, there were 300 LCs, 300 NCs, and 144 BLDs in the validation cohort, which was used to validate the TAAbs from the verification cohort. In phase III, the ELISA results of eight TAAbs of the LCs and NCs in validation cohort were applied to build and test the diagnostic models.

### Screening 12 Potential TAAbs for LC Based on Focused Protein Array

**Figure 2:**
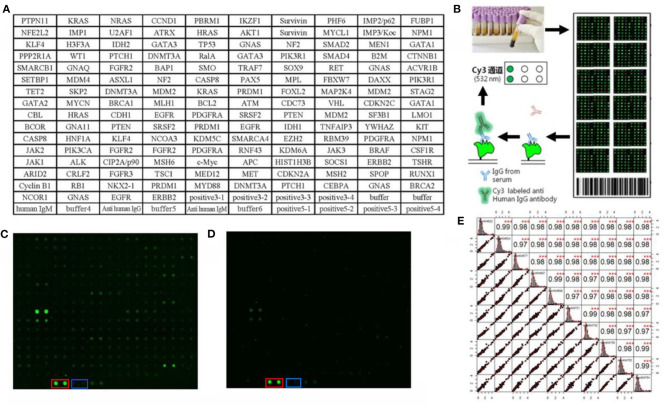
Protein array customization and preliminary results. **(A)** Protein array layout. **(B)** The operation process and principle of the protein array. **(C,D)** Protein fluorescence quantification results of LC and NC, respectively. The red and blue frames highlight the positive control (anti-human IgG) and negative control (buffer). **(E)** The result of repeated experiments by the same serum sample. The lower left showed the distribution of the results after linear fitting, and the upper right showed correlation results between samples after linear fitting (***P < 0.001). The middle graph was the cumulative density distribution of a single sample.

One hundred serum samples from LCs and 50 sera from NCs were tested by customized protein array. The 154 human recombinant protein, positive control (antihuman IgG) and negative control (buffer) arranged according to the protein array layout that shows in Figure 2A. The operation process and principle of the protein array were visualized in Figure 2B. As shown in Figures 2C,D, the fluorescent scanning signal results of two representative samples illustrated that the IgG response of the LC case was stronger than the NC.

Before the formal experiment, we repeated the tests 30 times in total on the same sample at different times, different arrays, and different locations to evaluate the stability of the array and the operation. From the results, the overall average value of repeatability between different batches of arrays was 0.98, indicating the overall stability was great (Figure 2E).

**Figure 3:**
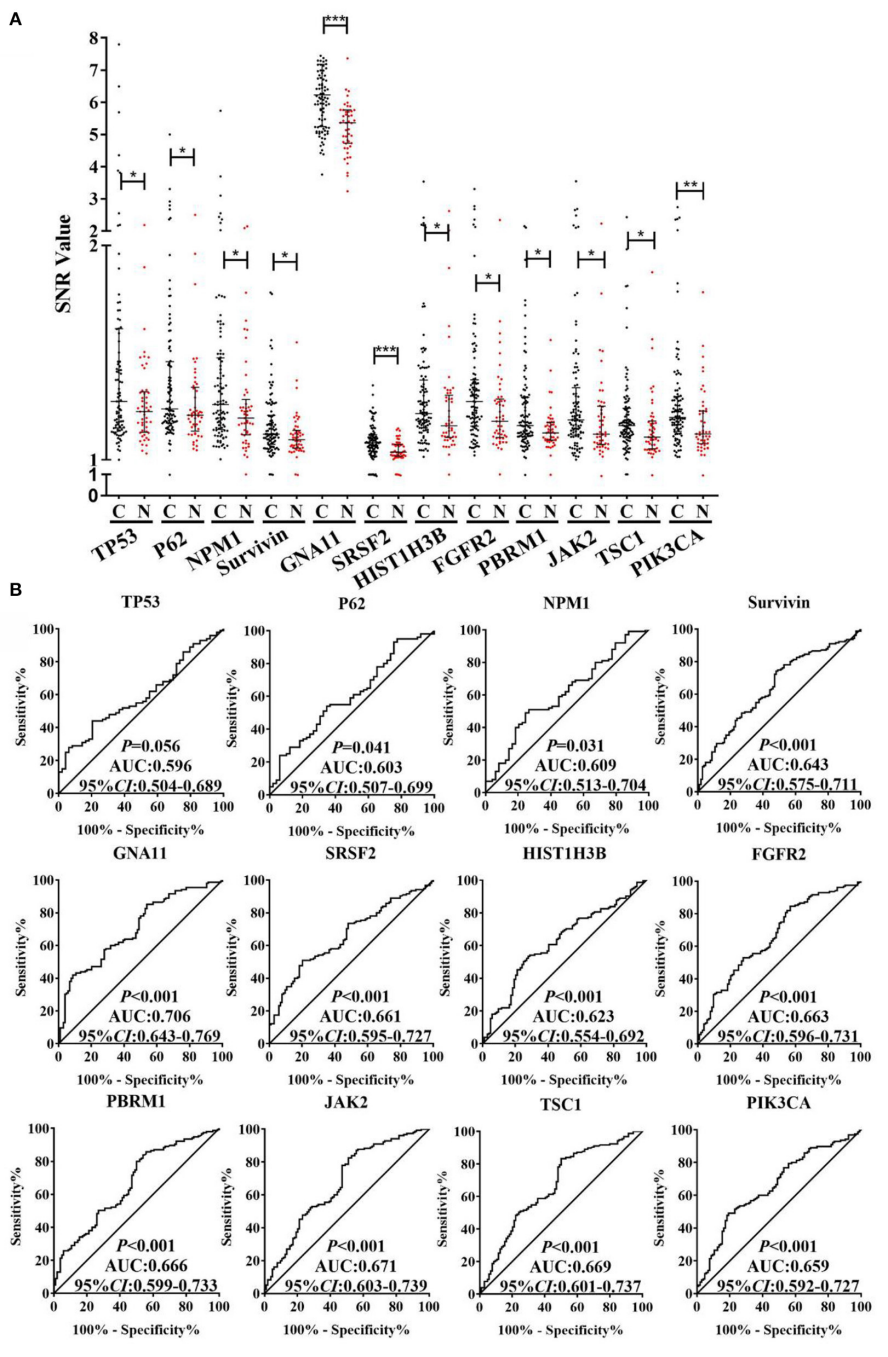
**(A)** SNR of autoantibodies against 12 TAAs in discovery cohort with 100 LCs and 50 NCs. **(B)** ROC analysis of autoantibodies against 12 TAAs for LC detection in discovery cohort. C, cancer; N, normal; ***P < 0.001; **P < 0.01; *P < 0.05.

As exhibited in the Supplementary Figure 1, based on the criteria of AUC >0.5 and *P* < 0.05 by ROC analysis, the 40 TAAbs were preliminarily screened (Supplementary Table 1). Then, totally 15 TAAbs of them were further screened, which included 11 TAAbs selected by regression analysis and four TAAbs studied in our previous research. Whereafter, according to the criteria of the positive rate of LC minus NC was > 10%, we ultimately selected 12 candidate TAAbs which involved in carcinogenesis, such as cell cycle, apoptosis, PI3K pathway, and RAS pathway (Supplementary Table 2) for further verification. Higher level of the 12 TAAbs was observed in LCs than NCs (*P* < 0.05) (Figure 3A). The AUC of each TAAb was ranged from 0.596 (95% CI: 0.504–0.689) to 0.706 (95% CI: 0.643–0.769) (Figure 3B).

### Verifying the Candidate TAAbs by ELISA in Verification Cohort

In order to determine the diagnostic validity of 12 TAAbs, we tested these TAAbs in 310 serum samples in the verification cohort (155 LCs and 155 NCs) by ELISA. The results were highly consistent with the discovery phase. According to screening criteria of AUC >0.5 and *P* < 0.05, four TAAbs (P62, Survivin, PBRM1, and JAK2) were excluded. The concentration level of the other eight TAAbs in the serum of LCs was significantly higher than NCs (*P* < 0.05) (Supplementary Figure 2A). As displayed in Supplementary Figure 2B, GNA11 owned the highest AUCof 0.802 (95% CI: 0.753–0.850).

### The Performance of the Eight TAAbs in Validation Cohort and Establishment of Diagnostic Model

**Figure 4:**
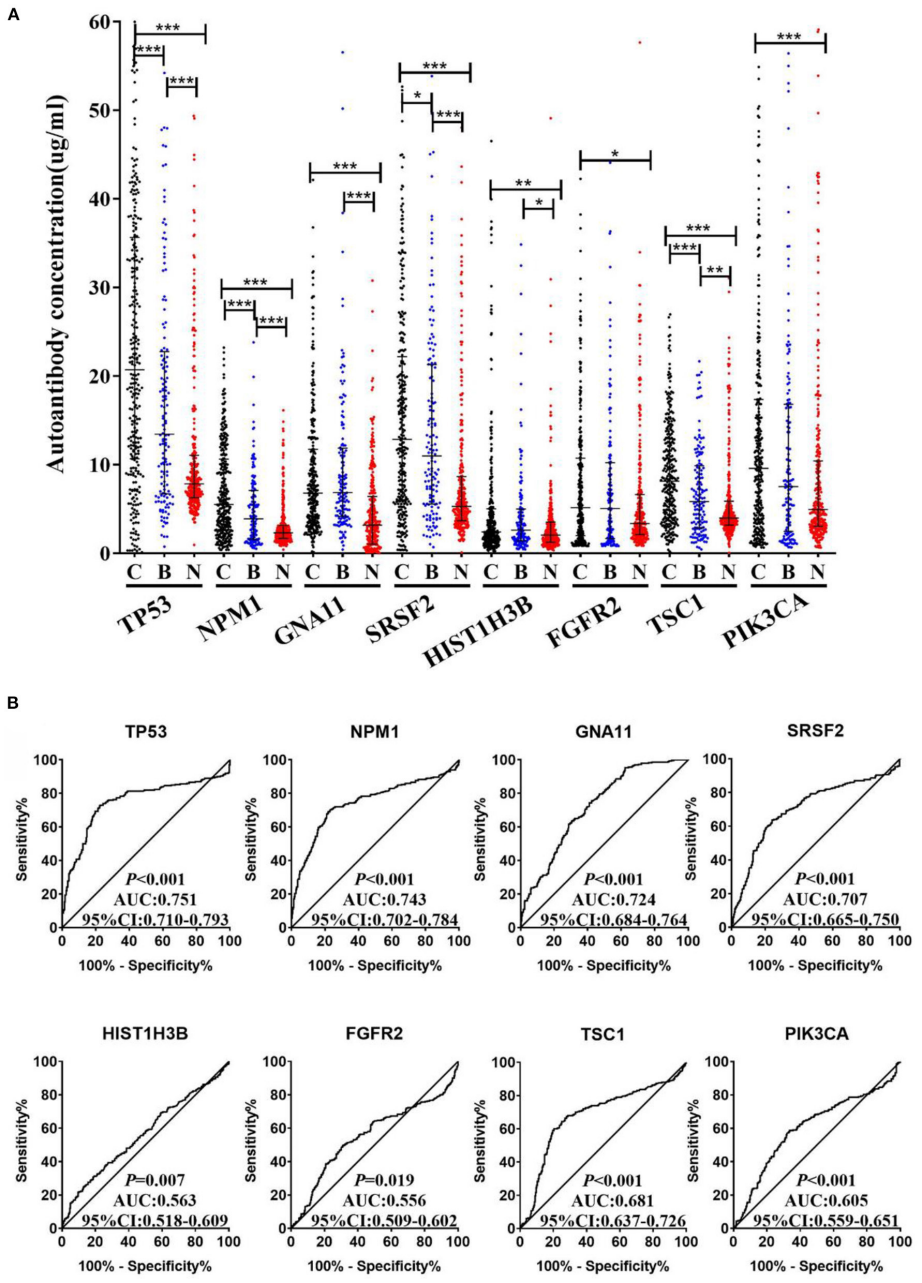
**(A)** The expression of autoantibodies against eight TAAs in validation cohort with 300 LCs, 144 BLDs, and 300 NCs. **(B)** ROC analysis of autoantibodies against eight TAAs for LC and NC groups in validation cohort. C, cancer; B, benign; N, normal; ***P < 0.001; **P < 0.01; *P < 0.05.

An independent validation cohort, including 300 LCs, 300 NCs, and 144 BLDs, was then used to validate the above eight TAAbs. As indicated in Figure 4A, all eight TAAbs showed significantly higher level in LCs compared with NCs. Interestingly, the serum levels of four TAAbs (TP53, NPM1, SRSF2, and TSC1) in LCs were significantly higher than BLDs. The AUCs of eight TAAbs for distinguishing LCs from NCs were ranged from 0.556 (95% CI: 0.509–0.602) for FGFR2 to 0.751 (95% CI: 0.710–0.793) for TP53 (Figure 4B), and the sensitivities were 13.7–43.0% at the specificities ≥90% (Supplementary Table 3). Besides, we investigated the correlation of the eight TAAbs and histologies; however, the results revealed that there were no differences among the adenocarcinoma patients, squamous cell carcinoma patients, and small cell lung cancer patients in serum TAAbs (*P* > 0.05) (data not shown).

**Figure 5:**
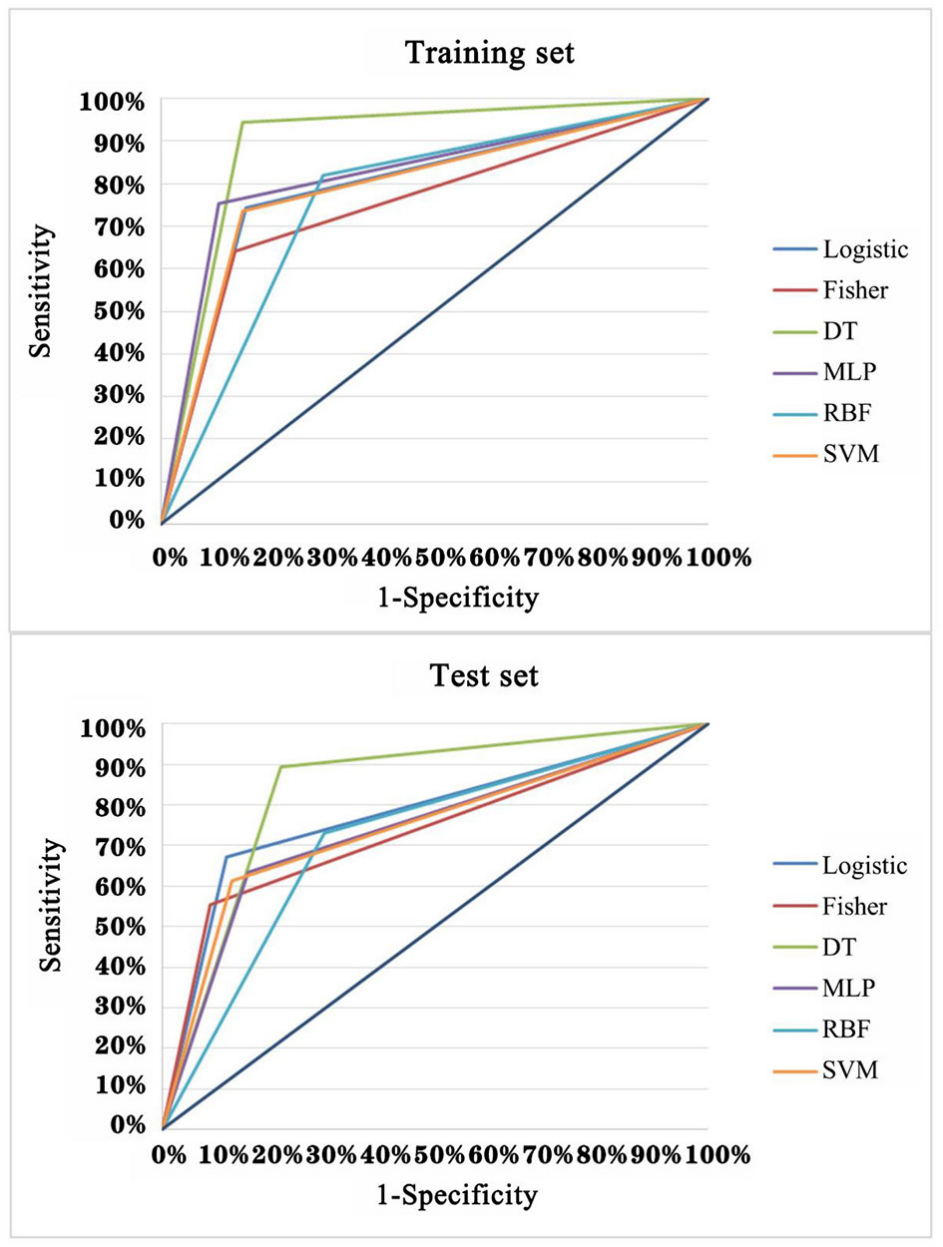
ROC analysis of multiple models for the differential diagnosis of LCs and NCs in training set and test set of validation cohort.

In order to explore the optimal diagnostic model with higher diagnostic accuracy than single TAAb for LCs, six modeling methods were performed and compared. Clearly, the model established by the DT C5.0 yield the most remarkable diagnostic performance among the six models (Figure 5), which contain seven TAAbs (TP53, NPM1, FGFR2, PIK3CA, GNA11, HIST1H3B, and TSC1) and possessed an AUC of 0.897 (95% CI: 0.863–0.924), sensitivity of 94.4%, specificity of 84.9%, and accuracy of 89.9% (Table 2). Meanwhile, it also achieved an excellent achievement in the test set, the AUC, sensitivity, specificity, and accuracy were 0.838 (95% CI: 0.777–0.888), 89.4, 78.2, and 83.3% (Table 2).

**Table 2:** The performance of multiple models in training set and test set for lung cancer detection.

**Modeling approach**	**TAAbs**	**Training set**					**Test set**				
		***P***	**AUC (95% CI)**	**Sensitivity (%) (95% CI)**	**Specificity (%) (95% CI)**	**Accuracy (%)**	***P***	**AUC (95% CI)**	**Sensitivity (%) (95% CI)**	**Specificity (%) (95% CI)**	**Accuracy (%)**
Fisher	5	<0.0001	0.753 (0.709–0.794)	64.2 (57.4–70.6)	86.4 (80.9–90.9)	74.9	<0.0001	0.732 (0.662–0.794)	55.3 (44.1–66.1)	91.1 (83.8–95.8)	74.7
Logistic	6	<0.0001	0.794 (0.752–0.832)	74.4 (68.0–80.1)	84.4 (78.6–89.2)	79.2	<0.0001	0.776 (0.709–0.834)	67.1 (56.0–76.9)	88.1 (80.2–93.7)	78.5
DT C5.0	7	<0.0001	0.897 (0.863–0.924)	94.4 (90.5–97.1)	84.9 (79.2–89.6)	89.9	<0.0001	0.838 (0.777–0.888)	89.4 (80.8–95.0)	78.2 (68.9–85.8)	83.3
MLP	8	<0.0001	0.824 (0.784–0.859)	75.4 (69.0–81.0)	89.5 (84.3–93.3)	82.1	<0.0001	0.738 (0.669–0.800)	63.5 (52.4–73.7)	84.2 (75.6–90.7)	74.7
RBF	8	<0.0001	0.761 (0.717–0.801)	81.9 (76.0–86.8)	70.4 (63.5–76.6)	76.3	<0.0001	0.716 (0.646–0.780)	72.9 (62.2–82.0)	70.3 (60.4–79.0)	71.5
SVM	8	<0.0001	0.792 (0.750–0.830)	73.5 (67.1–79.3)	84.9 (79.2–89.6)	79.0	<0.0001	0.742 (0.672–0.803)	61.2 (50.0–71.6)	87.1 (79.0–93.0)	75.3

*AUC, area under the receiver operating characteristic curve; CI, confidence interval; DT C5.0, Decision Tree C5.0; Fisher, Fisher discriminant analysis; Logistic, Logistic regression analysis; MLP, multilayer perception; RBF, radial basis function; SVM, support vector machines; TAAbs, autoantibodies to tumor-associated antigens; 5 TAAbs, TP53, NPM1, FGFR2, GNA11, and HIST1H3B; 6 TAAbs, TP53, NPM1, FGFR2, PIK3CA, GNA11, and HIST1H3B; 7 TAAbs, TP53, NPM1, FGFR2, PIK3CA, GNA11, HIST1H3B, and TSC1; 8 TAAbs, TP53, NPM1, SRSF2, FGFR2, PIK3CA, GNA11, HIST1H3B, and TSC1*.

### Evaluation of the Performance of the Optimal Model in Different Stages of LC

According to clinical stages I, II, III, and IV (AGCC), stages I and II of LC were defined as early LC (*N* = 72) and stages III and IV as late LC (*N* = 141) (Table 3). For the diagnosis of early LC, TP53 owned the highest AUC (95% CI) of 0.840 (0.782–0.898), while the AUC of DT C5.0 model achieved 0.886 (95% CI: 0.845–0.926). The sensitivity of single TAAb in early LC ranged from 13.9 to 48.6%, while it dramatically increased to 94.4% in DT 5.0 model established by seven TAAbs. However, the specificity of the model (82.7%) was slightly reduced compared with the single TAAb (92.0–95.3%). For the late LC, the AUC (95% CI), sensitivity of DT C5.0 model were 0.864 (0.826–0.902) and 90.1%, which were obviously higher than single TAAb. Yet, the specificity of the model was only reduced about 10% in late LC compared with the single TAAb. Moreover, the accuracies of the model in both early and late stages were close to 90%, which highly improved the results of single TAAbs.

**Table 3:** The diagnostic performance of DT 5.0 model and the seven TAAbs in early and late stage LC.

**TAAbs**	**AUC (95% CI)**	***P***	**Sensitivity (%)**	**Specificity (%)**	**YI**	**PPV (%)**	**NPV (%)**	**Accuracy (%)**
**Early stage (I + II;** ***N*** **= 72)**								
TP53	0.840 (0.782–0.898)	0.000	48.6	92.7	0.413	86.89	64.33	70.64
NPM1	0.837 (0.778–0.897)	0.000	48.6	94.0	0.426	89.01	64.65	71.31
GNA11	0.733 (0.672–0.793)	0.000	26.4	95.3	0.217	84.97	56.43	60.86
HIST1H3B	0.567 (0.484–0.650)	0.078	13.9	95.3	0.092	74.85	52.54	54.61
FGFR2	0.639 (0.558–0.719)	0.000	15.3	94.0	0.093	71.80	52.60	54.64
TSC1	0.749 (0.683–0.816)	0.000	18.1	92.0	0.101	69.30	52.89	55.03
PIK3CA	0.668 (0.592–0.744)	0.000	15.3	93.3	0.086	69.62	52.42	54.31
DT C5.0	0.886 (0.845–0.926)	0.000	94.4	82.7	0.771	84.49	93.70	88.56
**Late stage (III + IV;** ***N*** **= 141)**								
TP53	0.710 (0.651–0.769)	0.000	35.5	92.7	0.281	82.86	58.95	64.06
NPM1	0.707 (0.650–0.764)	0.000	27.0	94.0	0.210	81.79	56.27	60.48
GNA11	0.727 (0.679–0.774)	0.000	19.1	95.3	0.145	80.41	54.11	57.24
HIST1H3B	0.565 (0.506–0.624)	0.027	9.2	95.3	0.046	66.39	51.22	52.28
FGFR2	0.509 (0.448–0.571)	0.750	7.8	91.0	−0.012	46.43	49.67	49.40
TSC1	0.641 (0.582–0.701)	0.000	9.2	92.0	0.012	53.54	50.33	50.61
PIK3CA	0.576 (0.516–0.636)	0.010	14.9	90.0	0.049	59.82	51.40	52.45
DT C5.0	0.864 (0.826–0.902)	0.000	90.1	82.7	0.727	83.86	89.28	86.37

*AUC, area under the receiver operating characteristic curve; CI, confidence interval; DT C5.0, Decision Tree C5.0; LC, lung cancer; NPV, negative predictive value; PPV, positive predictive value; TAAbs, autoantibodies to tumor-associated antigens; YI, Youden's Index*.

## Discussion

In recent years, with the rapid development of proteomics methods, the discovery of new serum biomarkers has been greatly promoted by protein array which is a high-throughput method to screen specific antibody targets against protein samples (25). Hence, the protein array technique was selected for high-throughput screening in current research.

Although one study has utilized protein array to identify TAAbs for LC (26), our research design owned several novel features. First, the protein array was customized based on 138 cancer driver genes which were the key carcinogenic factors that could promote the rapid growth of tumors. On this basis, the possibility of screening out meaningful biomarkers was improved to some extent. Second, the candidate TAAbs were verified and validated in the multiple independent cohorts with more than 1,000 samples, so that the diagnostic value of these TAAbs was very reliable on account of the consistency between ELISA and protein array results. Third, we applied multiple data mining methods to establish diagnostic models and then selected the optimal model, which not only yielded further improvements in diagnostic performance but also avoided the insufficiency of using a single modeling approach.

Cancer is a disease that is caused by the DNA sequence in the genomes of cancer cells changing (20). Besides, cancer driver genes were defined as the important genes which related to the occurrence and development of cancer, and the determination of cancer driver genes is key to advancing diagnostics, therapeutics, and treatments (27). Bert Vogelstein et al. (21) summarized 138 cancer driver genes (74 tumor suppressor genes and 64 oncogenes) which can promote or “drive” tumorigenesis when altered by intragenic mutations. We customized a protein array including 154 human recombinant proteins based on the 138 genes to explore the level of autoantibodies to the proteins encoded by these genes, which integrated the merits of cancer driver gene and TAAb.

Applying the protein array technology, we analyzed the level of autoantibodies against 154 proteins in serum from 100 LCs and 50 NCs. According to multiple statistical analyses and screening criteria, 12 TAAb candidates were rapidly identified in the discovery phase. These TAAbs are all involved in some important carcinogenesis functions (Supplementary Table 2), and eight of them were first discovered in this research for diagnosis of LC. The remaining four TAAbs have been studied in various cancers, including TP53 (28–30), P62 (31, 32), NPM1 (33, 34), and Survivin (35).

In the verification phase, these 12 TAAbs were tested using indirect ELISA in 155 LCs and 155 matched NCs to assess their performance in distinguishing LCs from NCs. Furthermore, eight TAAbs (TP53, NPM1, GNA11, SRSF2, HIST1H3B, FGFR2, TSC1, and PIK3CA) were further selected on account of their excellent performance in verification cohort and subjected to validation cohort with 300 LCs, 300 NCs, and 144 BLDs. The basically consistent results of multistage and multicohort validation testified the reliability of our study. Remarkably, the level of anti-TP53 was found to be statistically significantly higher in LC than NC, which yielded the highest diagnostic value with the AUC (95% CI) of 0.751 (0.710–0.793). Park et al. (36) also found the significance of anti-TP53 in the diagnosis of LC. Besides, it was regrettably found that the majority single TAAbs had lower diagnostic performance for LC, which was similar to the results shown in previous studies (37). In order to improve the diagnostic value, we combined different TAAbs by using diverse data mining methods.

In recent years, various data mining techniques have been widely used to establish cancer diagnostic models, such as logistic regression analysis (38), Fisher discriminant analysis (39), decision tree (40), support vector machine (41), ANN-MLP, and ANN-RBF (42). However, each method has its own strengths and weaknesses, so the current study aimed to build LC diagnostic models through different modeling methods and validate the diagnostic value of each model for LC in a test set for choosing an optimal model. In result, we selected the decision tree model with a seven-TAAb panel (TP53, NPM1, FGFR2, PIK3CA, GNA11, HIST1H3B, and TSC1) which yield the highest AUCs of 0.897 (95% CI: 0.863–0.924) and 0.838 (95% CI: 0.777–0.888) for distinguishing LCs from NCs in training set and test set. Moreover, the results of the seven TAAbs and the panel of TAAbs in this study showed better discriminatory power for the early-stage LC than the advanced stage (Table 2). The above result may imply that autoantibodies to tumor-associated antigens, as a kind of promising biomarkers produced in early stage of tumorigenesis, could own more chances to be applied for early diagnosis in cancers.

However, as to the limitation, the small sample size of early-stage LCs might limit the expansibility of the value of this diagnostic model. Therefore, in our further research, we will confirm the diagnostic utility of this TAAb panel in a large sample size study to verify our findings, and explore its differential diagnostic performance between benign and malignant pulmonary nodules.

In conclusion, focused protein array based on cancer-driver genes is an effective and fast approach to discovering novel TAAbs. Comprehensive analysis of multiple models established by data mining showed that the DT C5.0 model generated by the combination of seven TAAbs had the highest LC diagnostic value. In consequence, the model may be the auxiliary means for clinicians to diagnose early-stage LC, and it may have a great influence in improving the accuracy of LC diagnosis.

## Data Availability Statement

The original contributions presented in the study are included in the article/Supplementary Material, further inquiries can be directed to the corresponding author/s.

## Ethics Statement

The studies involving human participants were reviewed and approved by Medical Ethics Committee of Zhengzhou University (Zhengzhou, China). The patients/participants provided their written informed consent to participate in this study. Written informed consent was obtained from the individual(s) for the publication of any potentially identifiable images or data included in this article.

## Author Contributions

LD and JZ: conception and design. LD: administrative support. TW, LP, CS, KW, and XW: provision of study materials or patients. DJ, XZ, ML, YW, PW, HY, and JS: collection and assembly of data. DJ and XZ: data analysis, interpretation, and manuscript writing. All authors: final approval of manuscript.

## Conflict of Interest

The authors declare that the research was conducted in the absence of any commercial or financial relationships that could be construed as a potential conflict of interest.

## Abbreviations

ANN-MLP: artificial neural network-multilayer perception
ANN-RBF: artificial neural network-radial basis function
AUC: area under the receiver operating characteristic curve
BLD: benign lung disease
CEA: carcinoembryonic antigen
CI: confidence internal
COPD: chronic obstructive pulmonary disease
CYFRA 21-1: cytokeratin-19 fragment
DT: decision tree
ELISA: enzyme-linked immunosorbent assay
LC: lung cancer
LDCT: low-dose computed tomography
NC: normal control
NSE: neuron-specific enolase
ROC: receiver operating characteristic
SEREX: serological analysis of recombination cDNA expression libraries
SERPA: serological proteome analysis
SNR: signal-to-noise ratio
SVM: support vector machines
TAA: tumor-associated antigen
TAAb: autoantibody to TAA.
