# Supplementary material for: Discovering Panel of Autoantibodies for Early Detection of Lung Cancer Based on Focused Protein Array
Source: Front Immunol. 2021 Apr 23;12:658922. doi: 10.3389/fimmu.2021.658922 (PMC8102818; doi:10.3389/fimmu.2021.658922)
Supplement: Supplementary file 1 [file Data_Sheet_1.DOCX]

**Supplementary text**

1. **Serum Specimen Preparation**

This study was approved by the Ethics Committee of Zhengzhou University (Zhengzhou, China), and all subjects provided written informed consent form. Five mL peripheral venous blood was drawn by blood collection tubes (contained inert separation adhesive and coagulant) from each participant who was in a state of fasting. Before stood at room temperature more than 2h, blood specimens were promptly sent to the laboratory with centrifugation at 3000rpm for 5min to separate the serum from the blood cells. Serum was extracted and equally divided into 500μl with 1.5mL Eppendorf tube by a disposable straw, then marked the Eppendorf tube with the date and number. Serum specimens were recorded on the registration form, and frozen at -80°C refrigerator. Repeated freezing and thawing was avoided before tested for protein array.

All blood samples of lung cancer patients (LCs) were drawn at the time of initial diagnosis by two experienced pathologists, before they received any treatment. Moreover, sera of normal controls (NCs) without pulmonary-related diseases or other cancers were collected from health examination populations in the same hospital. Benign lung disease cases (BLDs) with chronic obstructive pulmonary disease (COPD) or chronic bronchitis were diagnosed based on clinical diagnostic criteria. Detailed clinical information of all subjects were collected from the medical record management system of the hospital, including name, age, gender, smoking history, drinking history, family tumor history, clinical stage, histological pathology type, tumor size, lymph node metastasis, distant metastasis and the detection results of traditional tumor biomarkers.

1. **Inclusion Criteria of Subjects**

Inclusion Criteria of LCs:

1. Age ≥ 20 years and ≤ 90 years
2. First diagnosed as LC by histopathological diagnosis
3. Have not received medical radiotherapy, chemotherapy, drugs or surgery
4. Without history of autoimmune diseases

Inclusion Criteria of NCs:

1. Age ≥ 20 years and ≤ 90 years
2. Medical examination report showed no abnormality
3. Without lung-related diseases, autoimmune diseases and other tumor-related diseases

Inclusion Criteria of BLDs:

1. Diagnosed as benign lung disease by general pulmonary function test.
2. Without history of malignant tumor and autoimmune disease
3. **Protein Array test**

**3.1 Protocol of protein array test**

Customized focused protein array (BC-BIO, Foshan, China) contained 154 human recombinant proteins which purchased from CDI Laboratories (Mayaguez, USA). Fourteen serum samples could be detected in one human protein custom array simultaneously. Protein arrays were stored at -80°C until required.

The protein arrays were taken out for 30 minutes to room temperature and incubated with blocking buffer (3% BSA in PBS buffer with 0.1% Tween 20) for 3 hours. Serum samples were diluted with PBS containing 0.1% Tween 20 detergent (PBS-T) at a ratio of 1:50. A total of 200μl of diluted serum samples was incubated on each subarray and overnight at 4°C. After washed with PBS-T, Alexa Fluor 532 goat anti-human IgG (Jackson ImmunoResearch, West Grove, PA) diluted 1:1000 with PBS-T was added and incubated to detect bound autoantibodies. It was washed again with PBS-T and centrifugally dried at room temperature. The scan was performed using LuxScan 10K-A (CapitalBio Corporation, Beijing, China), and the captured fluorescence data was analyzed using GenePix Pro 6.0 software (Molecular Devices).

**3.2 Data extraction**

F532 Median was referred to signal strength, which is the median value of strength for all the pixel point in each signal point. B532 Median was referred to background value, which is the median value of strength for the pixels around each signal point within a certain range of the background. For the extracted data, F532 median/B532 median, the ratio of the foreground value to the background value of each protein point, was calculated in order to eliminate the deviation caused by the inconsistent background values between different samples. Signal to noise ratio (SNR) was defined as the average value of F median/B median from duplicative protein points. The further statistical analyses of samples were all based on SNR.

**3.3 Analysis of stability**

During the experiment, we repeated the tests on the samples at different times, different arrays, and different locations to evaluate the stability of the operation. Repeat the test 30 times in total, and the incubation array number and the result diagram was shown in **Figure 2E**. The lower left showed the distribution of the results after linear fitting, and the upper right showed correlation results between samples after linear fitting (*indicate significance *P* < 0.05). The middle graph was the cumulative density distribution of a single sample. From the results, the overall average value of repeatability between different batches of arrays was 0.98, indicating the overall stability of 532nm channel was great.

1. **Detailed Steps of the Indirect ELISA Experiment**

The 12 purified human source recombinant proteins from Cloud-Clone Corp were individually dissolved in corresponding protein diluents to a stock solution with the concentration of 0.25µg/µl. Moreover, the optimal coated concentrations of the 12 recombinant proteins (TP53, IMP2/p62, Survivin, NPM1, GNA11, HIST1H3B, SRSF2, FGFR2, PBRM1, PIK3CA, JAK2 and TSC1) were 0.5, 0.125, 0.5, 0.125, 0.5, 0.125, 0.125, 0.125, 0.125, 0.125, 0.125 and 0.125ug/ml, respectively. Firstly, each protein was diluted to the optimal concentration with coating buffer (50Mm sodium carbonate/ bicarbonate pH 9.6), and added 100μl to each well onto 96-well plates to incubate at 4˚C for overnight. Human IgG antibody (Solarbio) was diluted in coating buffer to final concentrations from 300ng/ml to 0ng/ml (300, 250, 200, 150, 100, 50, 10 and 0ng/ml) to generate standard curve for each plate. After blocked with 200μl 2% bovine serum albumin (BSA) overnight at 4˚C, phosphate-buffered saline (PBS) containing 0.05% Tween 20 (PBST) was used to wash the plates for three times. Serum samples were diluted at 1:100 and added 100μl into each well, then incubated for 1 h at 37˚C. The plates were washed five times and incubated in 100μl of the HRP-rec-Protein A which diluted with 1% BSA to 1:10000 at 37˚C for 1h. 3, 3’, 5, 5’-tetramethylbenzidine (TMB)-H_2_O_2_-urea was added as substrate solution after the plates were washed five times with PBST. 50μl 2M H_2_SO_4_ was added into each well to stop the reaction. Finally, the optical density (OD) values were obtained at the dual wavelength of 450nm and 620nm by using a microplate reader. The concentration of autoantibodies in the serum was calculated according to the IgG standard curve. Furthermore, the positive and negative control serums of each TAAb were set in each plate for quality control. All positive and negative control sera were screened from the preliminary experiments.

**Supplementary Figures and Tables**

**Table S1.** 40 TAAbs overexpressed in lung cancer of the discovery cohort

| ID | Name | AUC | *P* | Fold Change | *P*-Non parametric |
| --- | --- | --- | --- | --- | --- |
| JHU15218.P160A02 | GNA11 | 0.742 | 0.000 | 1.227 | 0.000 |
| JHU14049.P147F11 | SRSF2 | 0.690 | 0.000 | 1.040 | 0.000 |
| JHU11201.P191E07 | PIK3CA | 0.660 | 0.002 | 1.094 | 0.002 |
| JHU01288.P186A08 | EZH2 | 0.637 | 0.007 | 1.039 | 0.007 |
| JHU12404.P130G10 | MAP2K4 | 0.630 | 0.010 | 1.066 | 0.010 |
| JHU12013.P191G04 | FGFR2 | 0.629 | 0.011 | 1.095 | 0.011 |
| JHU11496.P120C11 | SKP2 | 0.629 | 0.011 | 1.064 | 0.011 |
| JHU13216.P139A10 | ATM | 0.628 | 0.011 | 1.056 | 0.011 |
| JHU18035.P223G03 | TSC1 | 0.624 | 0.014 | 1.046 | 0.014 |
| JHU00930.P010F04 | PBRM1 | 0.623 | 0.015 | 1.076 | 0.015 |
| JHU19983.P185G08 | SMARCA4 | 0.622 | 0.016 | 1.115 | 0.016 |
| JHU19049.P193B03 | RNF43 | 0.621 | 0.017 | 1.092 | 0.017 |
| JHU13959.P146A11 | PDGFRA | 0.620 | 0.017 | 1.104 | 0.017 |
| JHU11540.P121B05 | DNMT3A | 0.619 | 0.019 | 1.108 | 0.019 |
| JHU09483.P099A11 | SETBP1 | 0.614 | 0.024 | 1.051 | 0.024 |
| JHU12779.P134B09 | MYCN | 0.614 | 0.024 | 1.050 | 0.024 |
| JHU12826.P134C12 | BRCA1 | 0.614 | 0.024 | 1.089 | 0.024 |
| JHU17771.P220B10 | TSHR | 0.613 | 0.025 | 1.044 | 0.025 |
| JHU02792.P030H12 | Survivin | 0.612 | 0.027 | 1.046 | 0.027 |
| JHU01314.P014D07 | PHF6 | 0.612 | 0.027 | 1.078 | 0.027 |
| JHU06648.P190F01 | JAK2 | 0.610 | 0.029 | 1.098 | 0.029 |
| JHU14142.P148D03 | PTEN | 0.609 | 0.030 | 1.262 | 0.030 |
| JHU12012.P191G03 | FGFR2 | 0.609 | 0.031 | 1.124 | 0.031 |
| JHU02070.P189E12 | NPM1 | 0.609 | 0.031 | 1.130 | 0.031 |
| JHU04737.P050E05 | GNAS | 0.609 | 0.031 | 1.210 | 0.031 |
| JHU04548.P048G09 | H3F3A | 0.609 | 0.032 | 1.047 | 0.032 |
| JHU13529.P142F02 | GATA1 | 0.608 | 0.032 | 1.046 | 0.032 |
| JHU09985.P105C04 | ASXL1 | 0.608 | 0.033 | 1.047 | 0.033 |
| JHU18685.P215F05 | HIST1H3B | 0.606 | 0.036 | 1.054 | 0.036 |
| JHU13326.P140E08 | CDC73 | 0.606 | 0.036 | 1.047 | 0.036 |
| JHU11421.P119G03 | TET2 | 0.604 | 0.039 | 1.062 | 0.039 |
| JHU17282.P205C02 | ALK | 0.604 | 0.040 | 1.062 | 0.040 |
| JHU13707.P144D07 | CDH1 | 0.604 | 0.040 | 1.111 | 0.040 |
| JHU01384.P015G02 | IMP2/p62 | 0.603 | 0.041 | 1.122 | 0.041 |
| JHU00236.P003B03 | KRAS | 0.603 | 0.042 | 1.144 | 0.042 |
| JHU22054.P240C05 | RB1 | 0.603 | 0.042 | 1.023 | 0.042 |
| JHU19391.P197F12 | JAK3 | 0.601 | 0.046 | 1.099 | 0.046 |
| JHU18103.P224F03 | MED12 | 0.601 | 0.046 | 1.072 | 0.046 |
| JHU19463.P198B09 | CSF1R | 0.600 | 0.047 | 1.117 | 0.047 |
| JHU04788.P050D05 | TP53 | 0.596 | 0.056 | 1.690 | 0.056 |

AUC: Area under the receiver operating characteristic curve; TAAbs: Autoantibodies to tumor-associated antigens.

**Table S2.** Protein information of twelve TAAbs

| Gene | Gene Name | Classification | Process | Core pathway | Access No. |
| --- | --- | --- | --- | --- | --- |
| TP53 | tumor protein p53 | TSG | Cell Survival | Cell Cycle/Apoptosis; DNA Damage Control | P04637 |
| IMP2/p62 | Insulin-like growth factor 2 mRNA binding protein 2 | RNA-binding | RNA-binding | Nuclear Proteins | Q9Y6M1-6 |
| Survivin | survivin (Surv) | TSG/oncogene | Cell Survival | Cell Death; Kinases; Nuclear Proteins; Signal Transduction | O15392 |
| NPM1 | Nucleophosmin1 | TSG | Cell Survival | Cell Cycle/Apoptosis | P06748 |
| GNA11 | guanine nucleotide-binding protein subunit alpha-11 | oncogene | Cell Survival | PI3K; RAS; MAPK | P29992 |
| HIST1H3B | histone cluster 1, H3b | oncogene | Cell Fate | Chromatin Modification | P68431 |
| SRSF2 | serine/arginine-rich splicing factor 2 | oncogene | Cell Fate | Transcriptional Regulation | Q01130 |
| FGFR2 | fibroblast growth factor receptor 2 | oncogene | Cell Survival | PI3K; RAS ; STAT | P21802 |
| PBRM1 | Protein polybromo-1 (PB1); BRG1- associated factor 180 (BAF180) | TSG | Cell Fate | Chromatin Modification | Q86U86 |
| PIK3CA | Phosphatidylinositol 4,5-bisphosphate 3-kinase catalytic subunit alpha isoform | Oncogene | Cell Survival | PI3K | P42336 |
| JAK2 | Janus Kinase 2 | Oncogene | Cell Survival | STAT | O60674 |
| TSC1 | Tuberous Sclerosis Protein 1 | TSG | Cell Survival | PI3K | Q92574 |

TAAbs: Autoantibodies to tumor-associated antigens; TSG: Tumor Suppressor Gene.

**Table S3.** Diagnostic value of eight TAAbs for LC in validation cohort*****

| TAAbs | AUC  (95%*CI*) | *P* | Sensitivity (%) | Specificity (%) | YI | PPV (%) | NPV (%) | Accuracy  (%) |
| --- | --- | --- | --- | --- | --- | --- | --- | --- |
| TP53 | 0.751  (0.710-0.793) | 0.000 | 41.0 | 90.0 | 0.310 | 80.39 | 60.40 | 65.50 |
| NPM1 | 0.743  (0.702-0.784) | 0.000 | 43.0 | 90.0 | 0.330 | 81.13 | 61.22 | 66.50 |
| GNA11 | 0.724  (0.684-0.764) | 0.000 | 26.3 | 90.0 | 0.163 | 72.45 | 54.98 | 58.15 |
| SRSF2 | 0.707  (0.665-0.750) | 0.000 | 32.0 | 90.0 | 0.220 | 76.19 | 56.96 | 61.00 |
| HIST1H3B | 0.563  (0.518-0.609) | 0.007 | 21.7 | 90.3 | 0.120 | 69.11 | 53.56 | 56.00 |
| FGFR2 | 0.556  (0.509-0.602) | 0.019 | 13.7 | 90.3 | 0.040 | 58.55 | 51.13 | 52.00 |
| TSC1 | 0.681  (0.637-0.726) | 0.000 | 26.0 | 90.0 | 0.160 | 72.22 | 54.88 | 58.00 |
| PIK3CA | 0.605  (0.559-0.651) | 0.000 | 19.7 | 90.0 | 0.097 | 66.33 | 52.85 | 54.85 |

*compared to normal controls

AUC: Area under the receiver operating characteristic curve; *CI*: Confidence interval; LC: Lung cancer; NPV: Negative predictive value; PPV: Positive predictive value; TAAbs: Autoantibodies to tumor-associated antigens; YI: Youden’s index.

**
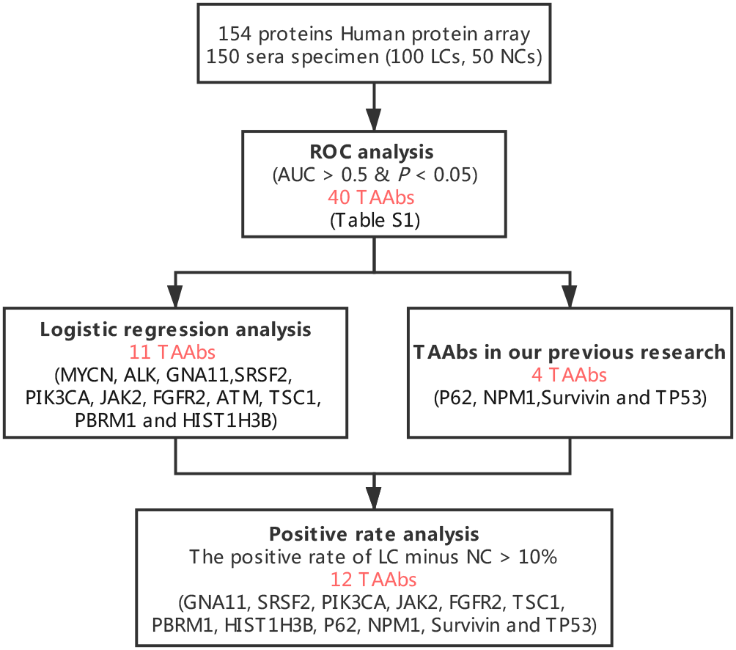
**

**Figure S1.** Flow chart of statistical screening methods for the initial screening of TAAbs based on the results of protein array.


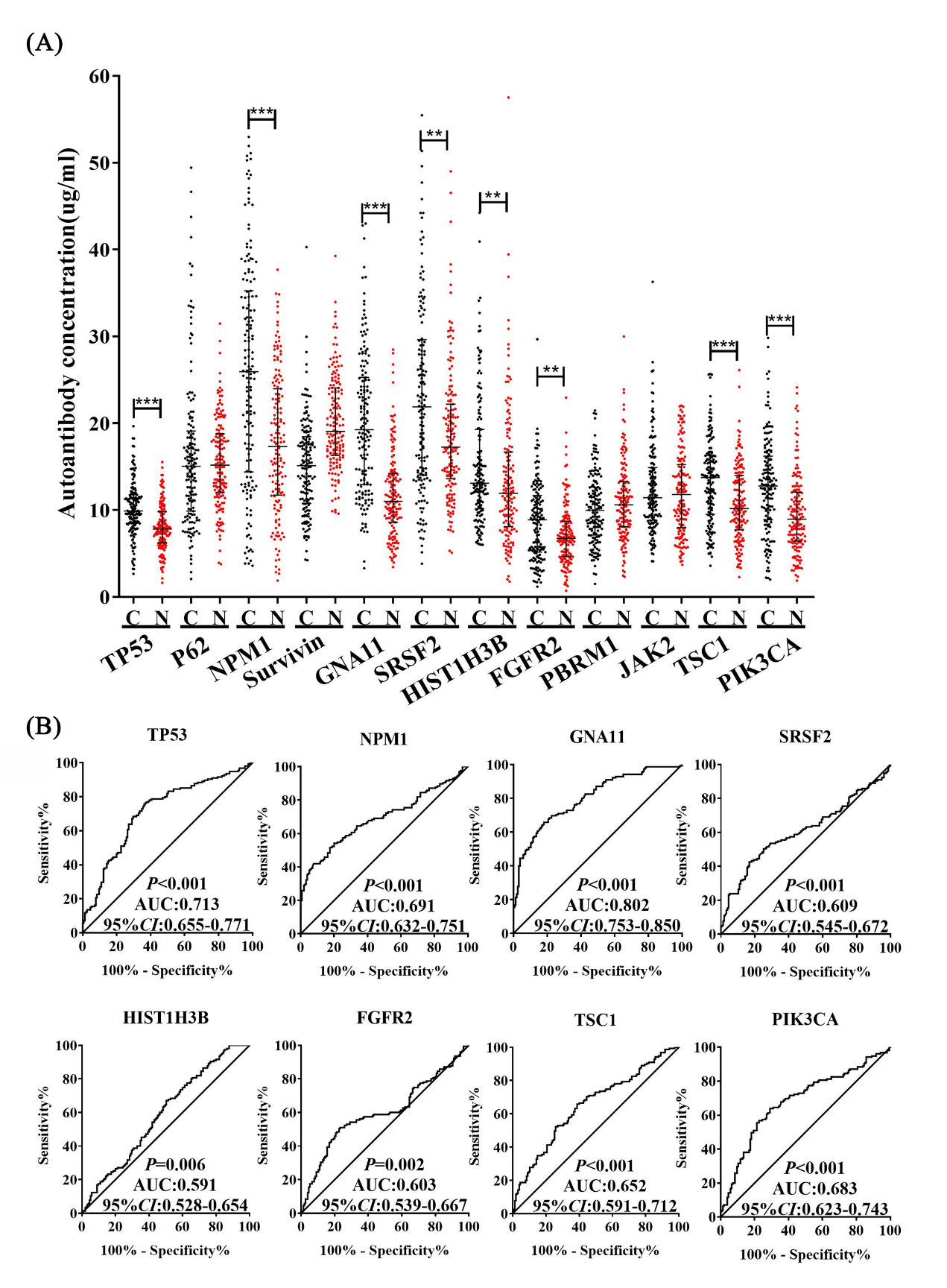


**Figure S2.** (A) Expression of autoantibodies against 12 TAAs in verification cohort with 155 LCs and 155 NCs. (B) ROC analysis of autoantibodies against 8 TAAs for LC detection in verification cohort. C: cancer; N: normal; ****P* < 0.001, ***P* < 0.01, **P* < 0.05.
